# The effects of run-of-river hydroelectric power schemes on invertebrate community composition in temperate streams and rivers

**DOI:** 10.1371/journal.pone.0171634

**Published:** 2017-02-03

**Authors:** Gary S. Bilotta, Niall G. Burnside, Matthew D. Turley, Jeremy C. Gray, Harriet G. Orr

**Affiliations:** 1 Aquatic Research Centre, School of Environment and Technology, University of Brighton, Brighton, United Kingdom; 2 Precima Inc., Toronto, Canada; 3 Environment Agency, Bristol, United Kingdom; University of Fribourg, SWITZERLAND

## Abstract

Run-of-river (ROR) hydroelectric power (HEP) schemes are often presumed to be less ecologically damaging than large-scale storage HEP schemes. However, there is currently limited scientific evidence on their ecological impact. The aim of this article is to investigate the effects of ROR HEP schemes on communities of invertebrates in temperate streams and rivers, using a multi-site Before-After, Control-Impact (BACI) study design. The study makes use of routine environmental surveillance data collected as part of long-term national and international monitoring programmes at 22 systematically-selected ROR HEP schemes and 22 systematically-selected paired control sites. Five widely-used family-level invertebrate metrics (richness, evenness, LIFE, E-PSI, WHPT) were analysed using a linear mixed effects model. The analyses showed that there was a statistically significant effect (*p*<0.05) of ROR HEP construction and operation on the evenness of the invertebrate community. However, no statistically significant effects were detected on the four other metrics of community composition. The implications of these findings are discussed in this article and recommendations are made for best-practice study design for future invertebrate community impact studies.

## Introduction

Renewable sources of energy are predicted to contribute almost one third of total electricity generation globally by 2035, with 50% of this from hydroelectric power (HEP) [1]. At present the greatest proportion of global HEP comes from large-scale storage-type schemes whereby rivers are dammed to create reservoirs [2]. The ecological effects of such large-scale storage schemes have been well-documented in the scientific literature [3–9]. In Europe, it is the knowledge of these potential impacts, together with the recognition that most opportunities for economically-profitable and politically-acceptable medium- to large-scale schemes have already been developed [10–11], that has caused attention to turn to smaller-scale HEP opportunities, particularly run-of-river (ROR) schemes, to help meet renewable energy and greenhouse gas emission targets [12].

Run-of-river HEP schemes are electrical power plants that use the flow within a river channel to generate electricity, without the need for water storage. A proportion of river flow is taken from the river (usually on a weir or a side channel), diverted down a secondary channel towards a HEP turbine, before being returned to the main channel further downstream [2, 12]. Such schemes vary in size; some larger installations located on major rivers can have power capacities of >1 MW, whereas the smallest ROR schemes on streams have power capacities of <10 kW. In Europe, there are relatively few multi-megawatt schemes; the majority of ROR schemes are mini (<1 MW) and micro (<100 kW) schemes installed on small river systems [12].

Run-of-river HEP schemes are often presumed to be less environmentally damaging than storage HEP schemes because they are normally built on, or make use of, existing weirs rather than involving the construction of large dams [11, 13]. However, whilst some research has demonstrated that the life-cycle water footprint [14], and life-cycle greenhouse gas emissions [6,15], from ROR HEP schemes can be significantly lower than those from large storage-type HEP schemes (and indeed most other sources of electricity), there is currently only limited evidence available to describe the ecological impact of these types of schemes [12,16].

A recent study [17] investigated the effects of ROR HEP schemes on fish community composition, using a multi-site Before-After Control-Impact (BACI) study design. The study found a small but statistically significant effect on the species richness of fish communities [17]. However, the conclusions were partially constrained by the low statistical power of the study, which was primarily associated with the relatively high spatial and temporal variability of fish populations, and the relatively low temporal resolution and accuracy of fish surveys [17]. The aim of this article is to investigate the effects of ROR HEP schemes on communities of invertebrates in temperate streams and rivers, using a similar multi-site BACI study design [18,19], that makes use of long-term routine environmental monitoring data collected according to standardised methods as part of national and international monitoring programmes.

This study is complementary to the previous study of the effects of ROR HEP schemes on fish communities; examining the effects of these schemes on another key component of freshwater ecosystems. Moreover, the monitoring of invertebrate communities offers a number of potential advantages that may improve the sensitivity and statistical power of environmental impact assessments compared to the monitoring of fish communities. Firstly, invertebrates are the most diverse group of freshwater organisms, communities of which can be comprised of many potential taxa, each with their own range of environmental tolerances/sensitivities [20,21]. Secondly, many invertebrates are often relatively sedentary and are therefore representative of site-specific ecological conditions [21], whereas the presence/absence of some migratory fish species can reflect the conditions of their marine habitats as much as their freshwater habitats. Thirdly, due to the relatively short life-span of most invertebrates, the communities respond relatively quickly to environmental changes [22], whereas for longer-lived fish species their presence can be sustained for more than a decade after the habitat became unsuitable for fish reproduction. Finally, owing to the relatively inexpensive sampling equipment and low-disturbance invertebrate sampling method, invertebrate monitoring programmes often involve more frequent sampling regimes than fish monitoring programmes, potentially improving the statistical power of impact assessments [18,19].

## Materials and methods

The methods used in this study broadly follow those described by Bilotta et al [17], though they are described in full within the following paragraphs for the purpose of precision and owing to differences associated with the use of invertebrate data and associated indices analysed in this study.

### Systematic search for operational ROR HEP schemes

The ROR HEP schemes included in this study were selected following a systematic search for HEP schemes, operating in England, which have meta-data available on their precise location, design, and dates of installation. There is no list that is publically-available for the UK that contains all of this information. However, England’s regulatory authority, the Environment Agency, collects information on proposed hydropower schemes when the developers apply for licences to abstract and/or transfer water from a river. This information provides a useful starting point for systematically identifying operational HEP schemes, but the limitations of this information are that not all schemes that are licenced get built, and the schemes that are built are not always constructed to the specifications detailed in the proposal. Furthermore, the information does not include a date of installation/commissioning, which is required to conduct a before-and-after analysis. Therefore independent verification of this licence information was required to confirm which of the proposed schemes have been built, what the final designs of the schemes entailed, and the dates that they became operational. This verification involved online searches (search engine: www.Google.co.uk) for the ‘name’ of the proposed scheme and the term ‘hydro’. If no relevant links were found within the first two pages of results on the search engine, then the scheme was deemed not operational. If some relevant links were found in the first two pages of search results for a scheme, then these were used to gather meta-data on the scheme, with further focussed online searches used where evidence suggested that a scheme was operational. This process does not necessarily produce an exhaustive list of operational schemes, but it is based on a systematic and transparent search. The search process identified 131 operational small-scale (< 5MW capacity) ROR HEP schemes in England out of the 313 schemes that applied for licences up until 31^st^ March 2014.

### Systematic identification of ROR HEP schemes with spatially and temporally co-located invertebrate monitoring

Once the operational ROR HEP schemes had been identified a proximity analysis was undertaken, in ArcGIS (v. 10.3), to identify which of the operational schemes had invertebrate monitoring surveillance sites located within a 1 km radius. In order to perform this analysis, the locations of all invertebrate monitoring surveillance sites in England, were extracted from the Environment Agency’s databases. Only samples collected by a standardised 3 min active kick sample technique were used. This technique, described by the Environment Agency [23], involves the use of a 900 μm mesh pond net to sample all in-stream habitats in proportion to their occurrence. A buffer tool (ArcGIS v10.3) was used to classify these features relative to operational HEP schemes, and the output selection set exported to Microsoft^®^ (MS) Excel. A subsequent manual visual check was then performed for each HEP scheme identified as having spatially co-located invertebrate data, using online mapping tools to ensure that the HEP scheme and the invertebrate monitoring site were indeed located on the same river. This step of the analysis included measurement of the approximate channel pathway distance between the HEP scheme and the invertebrate monitoring site, and recording whether the monitoring site is upstream or downstream of the HEP scheme. Finally, the dates of invertebrate monitoring were compared with the dates that the respective HEP scheme became operational, to ensure that the scheme had invertebrate monitoring data available for both the period before and after installation (referred to herein as temporal co-location).

**Fig 1:**
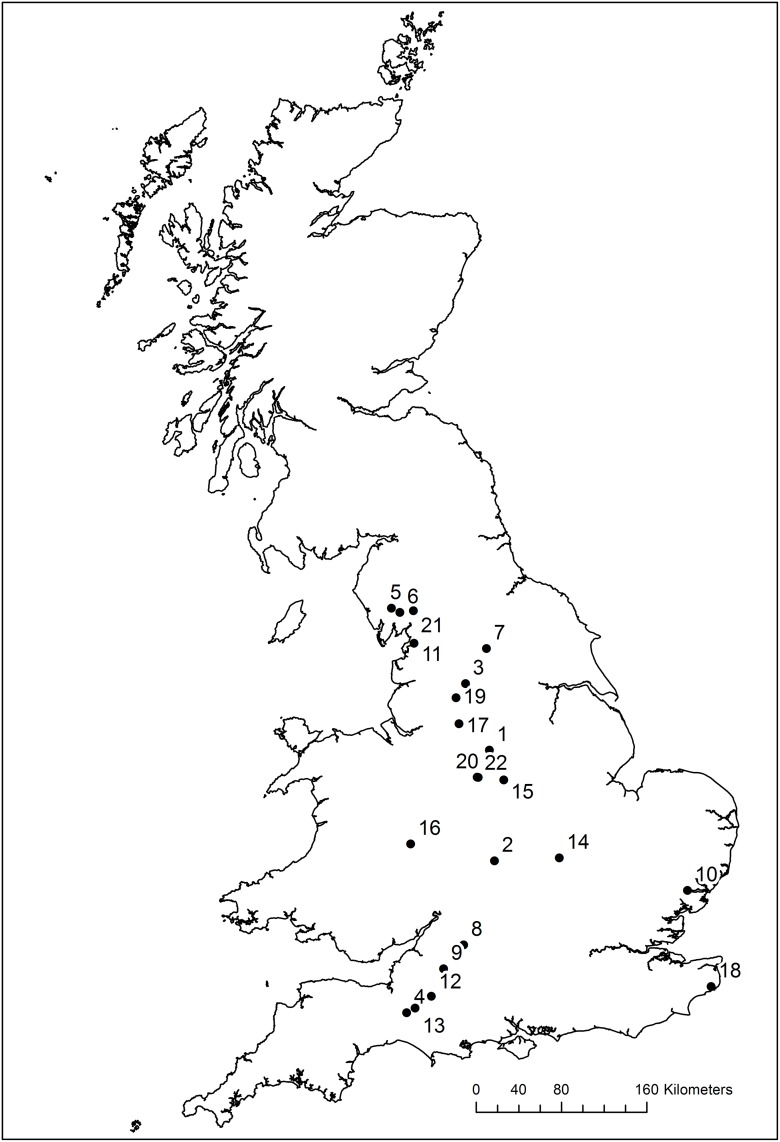
Locations of the 22 HEP schemes with spatially and temporally co-located invertebrate monitoring data. Reprinted from Ordnance Survey (Digimap Licence) under a CC BY license, with permission from Crown Copyright and Database Right [2016].

A total of 22 of the 131 verifiable operational HEP schemes in England had spatially and temporally co-located invertebrate monitoring surveillance data. As highlighted in S1 Table, the selected ROR HEP schemes incorporate a range of turbine designs (reverse-Archimedes screw, crossflow, Kaplan, Turgo, waterwheel), head heights (1–80 m; median of 2 m), and power capacities (4–314 kW; median of 25 kW). These schemes are fairly typical of ROR HEP schemes in Europe, and as illustrated in Fig 1, they also occur across a broad geographic area.

### Systematic identification of control sites with temporally co-located invertebrate monitoring

The invertebrate community response at HEP ‘impact’ sites were compared to those at respective ‘reference’ or ‘control’ sites that lack the influence of a HEP scheme, but that are: (1) local and therefore likely to have experienced similar fluctuations in weather and hydrological conditions (floods and droughts) over the corresponding period of monitoring of the ROR HEP ‘impact’ site, and (2) have been influenced by similar historical river management legacies, specifically, the presence of weirs. The second criterion was chosen because most English ROR HEP schemes are constructed on, or make use of, one of the 14,886 existing man-made weirs that are present in England [12,24]; as a result the invertebrate communities living near these schemes are unlikely to represent pristine or unaltered communities before the ROR HEP scheme is constructed. As such, the ‘control’ sites should also not start with pristine or unaltered communities [17]. The Environment Agency’s River Obstructions Database [24] provided the location and characteristics of weirs in England. The control sites were selected using a proximity analysis undertaken in ArcGIS (v. 10.3), to identify weirs within a 20 km radius of each operational ROR HEP scheme. A buffer tool was used to classify these features relative to operational ROR HEP schemes, and the output selection set exported to MS Excel.

Once all weirs within 20 km of the selected HEP scheme had been identified, a secondary proximity analysis was used to identify which of those weirs had invertebrate monitoring surveillance data within a 1 km radius. This proximity analysis used was identical to the process for identifying ROR HEP schemes with spatially and temporally co-located monitoring data (described above), including the subsequent manual visual check. In addition, the periods of invertebrate monitoring at each potential control site were compared with the period of invertebrate monitoring for the respective paired HEP scheme. For most HEP schemes, there were multiple potential control sites identified through the proximity analysis. In order to select a control site for each HEP scheme in a systematic manner, whilst minimising data processing time, the latter manual checks were conducted in an ascending order based on invertebrate monitoring site ID number. Once ten potential sites had been assessed (i.e. ten sites with spatially-co-located monitoring data covering the before and after period of the corresponding HEP scheme), then the process stopped and the invertebrate monitoring site with the greatest number of matched years of monitoring, with regards to the respective HEP scheme’s monitoring, was selected as the control site. This process resulted in the selection of 22 spatially and temporally co-located control sites. Meta-data on the period of monitoring and the number of invertebrate surveys at each of the 22 impact and 22 control sites is displayed in S2 Table.

### Data analysis

The null hypothesis of the study was that the construction and operation of ROR HEP schemes has no impact on the local (within ~1 km) invertebrate community composition. Two primary metrics of invertebrate community composition were analysed: (1) family richness, as a measure of total biodiversity, and (2) the Shannon-Wiener evenness index [25], as a measure of the ecological evenness (when there are similar proportions of all species, evenness approaches a value of 1; when the abundances are very dissimilar, then the value for evenness decreases towards 0). In addition, three diagnostic metrics of invertebrate community composition were analysed. These included the Lotic-invertebrate Index for Flow Evaluation (LIFE) [26], the Empirically-weighted Proportion of Sediment-sensitive Invertebrates (E-PSI) index [27], and the Walley-Hawkes-Paisley-Trigg (WHPT) index [28]. These diagnostic indices were selected as they are widely used in environmental status reporting and to underpin regulatory decisions about water resource management and environmental protection. The principles behind these diagnostic indices are summarised in the following paragraphs.

The LIFE score is designed to identify the degree of ecological change associated with flow modification in streams and rivers [26]. The LIFE score assigned to taxa is based on published quantified preferences and expert opinion regarding the sensitivity of benthic macroinvertebrates, at both species and family level, to flow velocity. An overall LIFE score is calculated for the sample from the sum of the individual species/family flow scores divided by the number of scoring species/families. LIFE scores lower than 6.00 generally indicate sluggish or still water conditions. As current velocity increases, so do LIFE scores. LIFE values higher than 7.5 indicate very fast flows. The index was therefore selected for inclusion in this study because of its capacity to detect potentially ecologically-significant changes to flow conditions that could occur as a consequence of HEP construction and operation.

The E-PSI index is a revised version of the Proportion of Sediment-sensitive Invertebrates (PSI) index that was designed to identify the degree of sedimentation in streams [29]. The PSI index was developed using a similar approach to LIFE, through assessment of invertebrate biological and ecological traits and previous literature. PSI assigned benthic macroinvertebrate taxa to different Fine Sediment Sensitivity Ratings (FSSR). The weighted relative abundance of FSSR groups (sensitive and insensitive) was used to calculate a PSI score; 0 being completely sedimented and 100 being unsedimented. In the E-PSI index, the macroinvertebrate taxa were empirically-weighted within the original classifications (sensitive/insensitive) to improve the final sediment-specificity of the index [27]. The index was therefore selected for inclusion in this study because of its capacity to detect potentially ecologically-significant changes to fine sediment that could occur as a consequence of HEP construction and operation.

The WHPT index [28] is a revised version of the original Biological Monitoring Working Party index that was designed to identify the degree of organic pollution [30]. The BMWP index was developed using a similar approach to LIFE through assessment of invertebrate faunal traits and previous literature. The BMWP system assigned benthic invertebrate taxa a score between 1 (tolerant to organic pollution) and 10 (intolerant to organic pollution). The BMWP score is the sum of the values for all families present in the sample. The number of BMWP-scoring families is typically recorded alongside the BMWP score, as is the Average Score Per Taxon (ASPT), which can be determined by dividing the BMWP score by the number of scoring taxa present. Although ASPT was primarily designed to indicate a response to organic pollution, it has also been used as an indicator of general degradation arising from human activities. Development of the WHPT index used empirical data to assign abundance related sensitivity weights to taxa. The taxa included in the index are modified from BMWP taxa; a number of taxa were removed due to insufficient data, some additional families were included where sufficient data were available, and some existing BMWP composite taxa were split into their constituent families. The WHPT-ASPT values typically range from 1 (indicative of sites with high organic pollution and degradation) to 13 (indicative of sites with very low organic pollution and degradation). The index was therefore selected for inclusion in this study because of its capacity to detect potentially ecologically-significant changes to water quality that could occur as a consequence of HEP construction and operation.

The hypothesis was tested, following the logic of beyond-BACI designs [18,19], by creating a linear mixed effect (LME) model, in the form of:

Response ~ BA * CI + (1|Year) + (1|Season) + (1|Site)

The terms Before-After and Control-Impact were modelled as fixed factors, while Calendar Year, Season and Site (to allow for paired control/treatment sites) were modelled as random effects. In this design, particular interest lies in the interaction (Before-After * Control-Impact), which, if significant, implies that the invertebrate communities of river sites with ROR HEP schemes (the impact group) responded differently to invertebrate communities of river sites without ROR HEP schemes (the control group). The statistical significance of the interactions was tested via an analysis of variance on the fitted models, using F statistics of the lmer function (lme4 library) available in free software (R 3.2.2). The *p*-values were calculated using the lmTest package within this software, with Satterthwaite approximation for degrees of freedom. Effect sizes were calculated using lsmeans, from the lsmeans package within this software. The proportional indices (Shannon-Wiener evenness index and E-PSI) were transformed prior to analysis, using a log(p/(1-p)) transformation [31].

## Results

**Fig 2:**
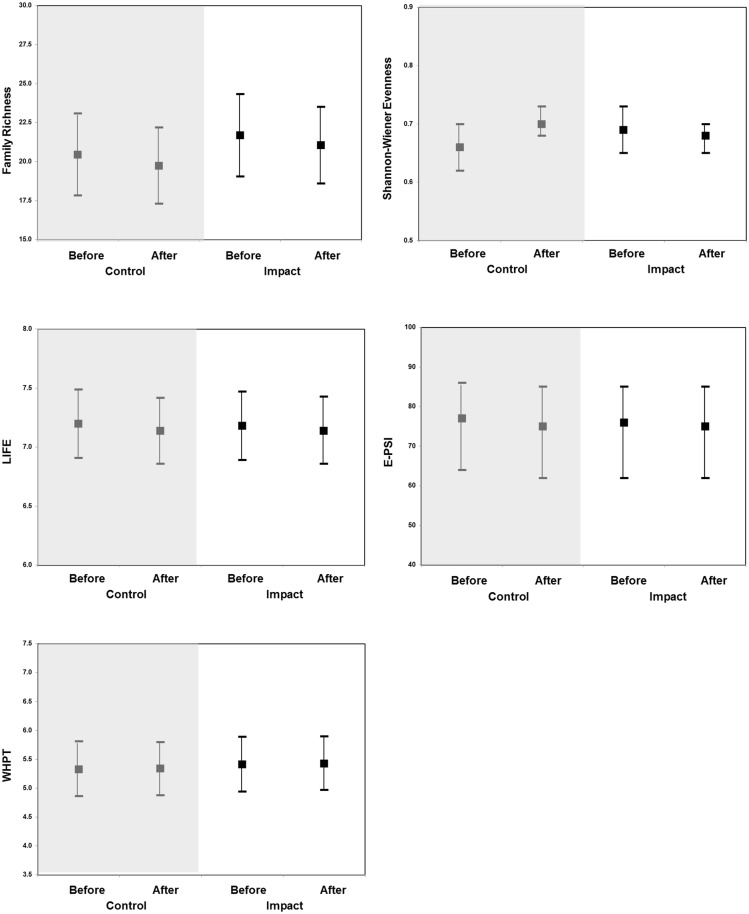
Fitted (least square) mean before and after values for the five invertebrate indices. Bars illustrate the upper and lower 95% confidence limits.

The fitted least squares means, standard errors, degrees of freedom, and 95% confidence limits for each treatment (Control-Impact) and period (Before-After), for the five invertebrate indices are shown in S3 Table and illustrated in Fig 2. As can be seen from Fig 2, there are only small changes in the mean values for each metric between the before period and after period in both the control and impact groups. There are also wide ranges for the upper and lower 95% confidence limits reflecting the variability of the metrics between sites within each group and within sites over time. Table 1 displays the BACI model outputs for the five invertebrate metrics.

**Table 1:** BACI model effect size and standard error (in parentheses) for the five invertebrate indices. Before-after contrast (B-A), control-impact contrast (C-I), before-after, control-impact interaction (BACI). Statistical significance: ** *p* <0.05 after Bonferroni-Holm correction for multiple comparisons. Note: The Shannon-Wiener evenness and E-PSI values are the transformed values.

	Dependent variable				
	Family richness	Shannon-Wiener evenness index	LIFE	E-PSI	WHPT-ASPT
B-A	-0.701 (0.651)	0.192** (0.070)	-0.057 (0.043)	-0.106 (0.093)	-0.005 (0.068)
C-I	1.231 (1.603)	0.140 (0.099)	-0.020 (0.106)	-0.066 (0.214)	0.077 (0.192)
BACI	0.065 (0.701)	-0.266** (0.099)	0.020 (0.046)	0.069 (0.103)	0.016 (0.070)
Observations	1465	1461	1463	1342	1465

Meta-data on the period of monitoring and the number of invertebrate surveys at each of the 22 impact and 22 control sites is displayed in S2 Table. The average distance between the HEP turbines and the invertebrate monitoring sites was 409 metres (median 305 metres). The average period of monitoring before construction of the 22 HEP schemes was 224 months (i.e. >18 years), with an average of 24 invertebrate surveys conducted per site during this period. The average period of monitoring after the construction of HEP schemes was 46 months (i.e. >3 years), with an average of 5 invertebrate surveys conducted per site during this period. The corresponding periods of monitoring for the control sites, were similar; average period of 246 months with an average of 30 invertebrate surveys conducted per site during the ‘before’ period, and an average monitoring period of 56 months, with an average of 7 surveys conducted per site during the ‘after’ period.

The invertebrate community metrics for each survey can be found in S4 Table. The five invertebrate indices studied exhibited substantial variability both among sites and over time within sites. Part of this temporal variation is likely to be associated with natural biological cycles and stochastic events, whilst part of the variation may also be associated with the precision of the sampling and sorting technique and taxa-identification. The resultant variability of the metrics influences the power of statistical tests (i.e. probability of correctly detecting a statistically-significant effect when one exists). Statistical power analysis of this study, according to the method described by Stroup [32], revealed that the magnitudes of BACI effect for which this study had an 80% chance of detecting (i.e. statistical power of 0.8) were: 1.88 for family richness, 0.06 for Shannon-Wiener evenness, 0.12 for LIFE, 5.54 for E-PSI, and 0.19 for WHPT-ASPT.

## Discussion

This study investigated the effects of ROR HEP schemes on communities of invertebrates in temperate streams and rivers, using a multi-site BACI study design that makes use of routine environmental surveillance data collected according to standardised methods as part of national and international monitoring programmes. The 22 ROR HEP schemes included in this study were systematically-selected, as were their paired control sites, which were located within a 20 km radius of their respective ROR HEP schemes, but on independent streams/rivers that also had the influence of management legacies (specifically the presence of weirs). The average period of monitoring before (>18 years) and after construction (>3 years) for each ROR HEP scheme is far greater than is normally possible through monitoring commissioned from standard academic research funding or short-term investigative studies.

The BACI effect size estimates are small for all five metrics of invertebrate community composition, with the 95% confidence intervals ranging from negative to positive for all of the metrics (i.e. an inconclusive effect). The construction and operation of ROR HEP schemes is estimated to have a very small positive effect on the family richness metric (0.07 more families post-construction), though the 95% confidence interval for the estimate of effect encompasses a range of -1.31 to 1.44. The construction and operation of ROR HEP schemes is estimated to have a very small negative effect on the Shannon-Wiener evenness index (back-transformed effect size of -0.06), indicating a slight reduction in community evenness post-installation. The 95% confidence interval for this estimate of effect ranged from -0.12 to 0.01. However, it should be noted that back-transformed confidence intervals such as those for Shannon-Wiener evenness have lower accuracy owing to the underlying non-linearity of standard errors for transformed variables. The construction and operation of ROR HEP schemes is estimated to have a very small positive effect on the LIFE metric (0.02), potentially indicating that the community composition has altered to reflect one adapted to marginally higher flows post-construction, though the 95% confidence interval for the estimate of effect encompasses a range of -0.07 to 0.11. The construction and operation of ROR HEP schemes is estimated to have a very small positive effect on the E-PSI metric (1.3 units), potentially indicating that the community composition has altered to reflect one adapted to marginally lower fine sediment deposition post-installation. The 95% confidence interval for the estimate of effect encompasses a range of -0.04 to 2.56, but once again it should be noted that back-transformed confidence intervals have lower accuracy owing to the underlying non-linearity of standard errors for transformed variables. The construction and operation of ROR HEP schemes is estimated to have a very small positive effect on the WHPT metric (0.02 units), potentially indicating that the community composition has altered to reflect one adapted to marginally improved water quality post-installation, though the 95% confidence interval for the estimate of effect encompasses a range of -0.12 to 0.15.

There was only one metric of invertebrate community composition, Shannon-Wiener evenness index, for which the BACI effect is statistically significant (p <0.05). In river sites with ROR HEP schemes, there was a very small decrease in mean evenness in the after-construction period relative to the before-construction period (0.69 before; 0.68 after). In contrast, in control sites there was a very small increase in mean evenness in the after-construction period relative to the before-construction period (0.66 before; 0.70 after). Although some researchers suggest that decreases in Shannon-Wiener evenness values can indicate environmental stress, it is important to note that values for this metric can also increase in the presence of environmental stress [33]. Furthermore, in this study, none of the diagnostic indices (LIFE, E-PSI, WHPT), suggested stressor-specific changes of a magnitude which would be of ecological concern. Nevertheless, these findings may warrant further investigation to establish the likely mechanisms of community composition change and to better understand longer-term trends in community composition.

With any inferential statistical test there is always the possibility that when a difference does exist, the test will not be able to identify it. This type of mistake is called a Type II error [34]. The statistical power of a test refers to the probability of making a Type II error. It is generally accepted that statistical power should be 0.8 or greater; that is, studies should have an 80% or greater chance of finding a statistically significant difference when one exists. However, consideration and reporting of statistical power is rare in environmental science studies. A review of research papers published in *Conservation Biology* and *Biological Conservation* that reported the results of null-hypothesis tests [35], showed that 92% of articles did not report statistical power. In addition, 63% of authors misinterpreted statistical non-significance as evidence for no effect [35]. Whilst it can be difficult to achieve a power of 0.8 in environmental studies and other disciplines [36], it is better for authors to acknowledge what their power was, rather than to ignore it. The results of studies with low statistical power can be both misleading and dangerous, not only because of their inability to detect ecologically significant changes, but also because they create the illusion that something useful has been done [37,38]. Statistical power analysis for this study revealed that the magnitudes of BACI effect sizes for which this study had an 80% chance of detecting (i.e. statistical power of 0.8) were 1.88 for family richness, 0.06 for Shannon-Wiener evenness, 0.12 for LIFE, 5.54 for E-PSI, and 0.19 for WHPT-ASPT. The estimated magnitudes of effect were smaller than these thresholds for four of these metrics, and therefore the statistical power for each of these four metrics was relatively low (0.05 for the family richness metric, 0.07 for the LIFE metric, 0.11 for the E-PSI index, and 0.06 for the WHPT-ASPT index). For prospective studies the statistical power and sensitivity could be increased through an increased number of surveys at each site, in addition to an increased number of sites within the study. The data used here were assembled opportunistically, and suffer from statistical noise associated with a variable temporal sampling effort, and also variance in seasons, site effects and years pre- and post- treatment. By designing sampling with statistical analysis in mind [19], these external effects can be minimized, and sampling effort can be more efficiently allocated. Future research should take this statistical power analysis into consideration when attempting to design studies to detect the impacts of interventions on invertebrate communities in temperate streams and rivers.

In this study different types of ROR HEP schemes were grouped together, regardless of design features such as turbine type, capacity, or head height. The authors recognise that different scheme designs may have different biological impacts, but we were not able to conduct any sub-analysis owing to the limited number of replicates of each scheme design and the limited statistical power. The effects observed are the average response monitored a median distance of 305 m upstream or downstream from ROR turbines with a median scheme capacity of 25 kW. The effects may vary with proximity to the ROR HEP schemes, and in particular between depleted and non-depleted reaches, but this could not be investigated using this retrospective study design. It may be possible to conduct a follow-on BACI study with a sub-analysis for ROR HEP scheme design, if it is possible to add data from further ROR HEP schemes with paired controls that have been monitored in a similar manner by regulatory authorities within other countries. However, it should be recognised that the results may be regulation-specific, and that most of the ROR HEP schemes included in this study have been developed in accordance with best-practice guidance from the regulatory authority of England [39–41]. This guidance details the regulatory requirements stipulating where/when it is necessary to install a fish pass, to include fish screens, and/or to halt abstraction/operation of the ROR HEP scheme during low flows. For ROR HEP schemes built in countries with a significantly different set of regulatory requirements, the effects of the schemes may be dissimilar to those observed in this study.

## Supporting Information

S1 Table Meta-data on each HEP scheme. (DOCX)

S2 Table Meta-data on invertebrate monitoring for each impact and control site. Asterisks indicate that invertebrate monitoring is within the depleted reach of a HEP scheme.
(DOCX)

S3 Table Least squares (LS) mean and 95% confidence limit (CL) for each treatment (Control-Impact) and period (Before-After), for the five invertebrate metrics. (DOCX)

S4 Table Invertebrate metrics for all sites and surveys. (XLSX)
