# Supplementary material for: The effects of run-of-river hydroelectric power schemes on invertebrate community composition in temperate streams and rivers
Source: PLoS One. 2017 Feb 3;12(2):e0171634. doi: 10.1371/journal.pone.0171634 (PMC5291416; doi:10.1371/journal.pone.0171634)
Supplement: S1 Table — (DOCX) [file pone.0171634.s001.docx]

**S1 Table. Meta-data on each HEP scheme**

| **HEP Scheme** | **Turbine Type** | **Capacity (kW)** | **Head (m)** | **Construction Start Date** | **Layout** | **Fish pass constructed as part of development** | **Hands-off flow (Q percentile)** |
| --- | --- | --- | --- | --- | --- | --- | --- |
|  |  |  |  |  |  |  |  |
| 1 | Crossflow | 30 | 4.2 | 12/2008 | 75m depleted reach | None | Q95 |
| 2 | Kaplan | 15 | 1.6 | 01/2014 | 17m depleted reach | Eel pass | Q95 |
| 3 | Archimedes | 10 | 2.2 | 05/2012 | 10m depleted reach | Existing fish pass | Q95 |
| 4 | Kaplan | 6 | 1.8 | 10/2005 | 80m depleted reach | None | N/A |
| 5 | Turgo | 314 | 80.2 | 07/2006 | 700m depleted reach | None | N/A |
| 6 | Crossflow | 45 | 10.0 | 02/2014 | 180m depleted reach | Fish and elver pass | Q85 |
| 7 | Waterwheel | 11 | 4.5 | 06/2009 | 295m depleted reach | None | N/A |
| 8 | Archimedes | 12 | 1.8 | 12/2009 | 75m depleted reach | None | Q95 |
| 9 | Archimedes | 55 | 1.8 | 01/2012 | 240m depleted reach | Larinier fish pass | Q90 |
| 10 | Archimedes | 11 | 1.1 | 11/2011 | 50m depleted reach | Larinier fish pass | Q95 |
| 11 | Kaplan | 100 | 2.1 | 06/2014 | 25m depleted reach | Larinier fish pass | Q90 |
| 12 | Crossflow | 12 | 4.8 | 10/2003 | 265m depleted reach | None | N/A |
| 13 | Archimedes | 23 | 1.6 | 12/2008 | 10m depleted reach | None | N/A |
| 14 | Archimedes | 15 | 1.2 | 12/2012 | 15m depleted reach | Eel pass | Q95 |
| 15 | Kaplan | 230 | 2.5 | 07/2012 | 40m depleted reach | Alaskan fish pass | Q95 |
| 16 | Kaplan | 165 | 1.5 | 05/2012 | 15m depleted reach | Larinier fish pass | Q95 |
| 17 | Archimedes (x2) | 68 | 1.4 | 04/2012 | 5m depleted reach | Fish ladder | Q95 |
| 18 | Crossflow (x3) | 3.6 | 2.6 | 07/2007 | 15m depleted reach | None | Q98 |
| 19 | Archimedes | 20 | 1.5 | 09/2010 | 10m depleted reach | Larinier fish pass | Q95 |
| 20 | Archimedes | 26 | 2.3 | 03/2010 | 1450m depleted reach | Larinier fish pass at the weir, Alaskan fish pass at the turbine | Q90 |
| 21 | Archimedes | 100 | 3.5 | 02/2013 | 30m depleted reach | Larinier | Q90 |
| 22 | Archimedes | 58 | 2.4 | 12/2009 | 25m depleted reach | None | None |
