# Supplementary material for: The effects of run-of-river hydroelectric power schemes on invertebrate community composition in temperate streams and rivers
Source: PLoS One. 2017 Feb 3;12(2):e0171634. doi: 10.1371/journal.pone.0171634 (PMC5291416; doi:10.1371/journal.pone.0171634)
Supplement: S2 Table — Asterisks indicate that invertebrate monitoring is within the depleted reach of a HEP scheme. (DOCX) [file pone.0171634.s002.docx]

**S2 Table. Meta-data on invertebrate monitoring for each impact and control site. Asterisks indicate that invertebrate monitoring is within the depleted reach of a HEP scheme.**

| **Scheme** | **Monitoring site** | **Latitude** | **Longitude** | **Distance (m) of monitoring upstream (US) or downstream (DS) from HEP turbine (or weir for control sites)** | **Period of monitoring before construction started (months)** | **Number of surveys before construction started** | **Period of monitoring after construction started (months)** | **Number of surveys after construction started** |
| --- | --- | --- | --- | --- | --- | --- | --- | --- |
|  |  |  |  |  |  |  |  |  |
|  |  |  |  |  |  |  |  |  |
|  |  |  |  |  |  |  |  |  |
|  |  |  |  |  |  |  |  |  |
| 1 | CONTROL | 53.04931 | -1.8762343 | 1000m DS | 279 | 50 | 81 | 9 |
|  | IMPACT | 53.1794 | -1.67228 | 310m US | 241 | 56 | 88 | 17 |
| 2 | CONTROL | 52.09421 | -1.6277363 | 1000m DS | 306 | 26 | 8 | 2 |
|  | IMPACT | 52.24626 | -1.60801 | 350m DS | 343 | 40 | 8 | 2 |
| 3 | CONTROL | 53.84216 | -1.8440343 | 400m DS | 266 | 29 | 16 | 2 |
|  | IMPACT | 53.73849 | -2.00754 | 825m DS | 266 | 33 | 28 | 4 |
| 4 | CONTROL | 51.1346 | -2.7122749 | 890m US | 127 | 7 | 115 | 6 |
|  | IMPACT | 50.96495 | -2.79127 | 125m DS | 121 | 10 | 108 | 7 |
| 5 | CONTROL | 54.29798 | -2.8969473 | 120m US | 137 | 16 | 98 | 8 |
|  | IMPACT | 54.36911 | -3.07586 | 740m DS | 136 | 17 | 87 | 6 |
| 6 | CONTROL | 54.25506 | -3.0652171 | 245m DS | 226 | 22 | 7 | 2 |
|  | IMPACT | 54.33419 | -2.95345 | 35m DS | 226 | 21 | 7 | 2 |
| 7 | CONTROL | 54.04406 | -1.9404062 | 20m DS | 230 | 46 | 82 | 14 |
|  | IMPACT | 54.03383 | -1.70683 | 25m DS | 225 | 13 | 71 | 4 |
| 8 | CONTROL | 51.43865 | -2.000282 | 560m US | 176 | 10 | 76 | 1 |
|  | IMPACT | 51.53868 | -2.03172 | 300m DS | 177 | 7 | 47 | 2 |
| 9 | CONTROL | 51.49262 | -2.2304128 | 590m US | 202 | 14 | 45 | 6 |
|  | IMPACT | 51.33825 | -2.30213 | 920m DS | 200 | 10 | 21 | 2 |
| 10 | CONTROL | 51.89448 | 0.86559585 | 240m DS | 300 | 49 | 46 | 8 |
|  | IMPACT | 51.95916 | 1.019372 | 100m US | 301 | 37 | 46 | 4 |
| 11 | CONTROL | 53.97493 | -2.5294455 | 495m US | 253 | 50 | 4 | 1 |
|  | IMPACT | 54.076 | -2.74568 | 10m DS | 241 | 41 | 15 | 3 |
| 12 | CONTROL | 51.21181 | -2.677847 | 290m US | 102 | 5 | 143 | 23 |
|  | IMPACT | 51.10663 | -2.46345 | 240m US | 103 | 6 | 72 | 7 |
| 13 | CONTROL | 51.16024 | -2.6490191 | 295m US | 165 | 8 | 70 | 6 |
|  | IMPACT | 51.00541 | -2.67979 | 620m DS | 127 | 4 | 59 | 8 |
| 14 | CONTROL | 52.31621 | -0.67818757 | 2035m US | 283 | 47 | 40 | 3 |
|  | IMPACT | 52.2645 | -0.71782 | 55m DS* | 283 | 46 | 11 | 2 |
| 15 | CONTROL | 52.80176 | -1.2609804 | 980m DS | 330 | 48 | 14 | 2 |
|  | IMPACT | 52.92655 | -1.47486 | 520m US | 317 | 24 | 44 | 3 |
| 16 | CONTROL | 52.35913 | -2.8786778 | 15m US | 293 | 42 | 47 | 9 |
|  | IMPACT | 52.38766 | -2.76255 | 750m US | 311 | 45 | 47 | 3 |
| 17 | CONTROL | 53.30432 | -2.3406897 | 245m DS | 314 | 41 | 19 | 2 |
|  | IMPACT | 53.40103 | -2.09712 | 75m DS | 305 | 24 | 42 | 9 |
| 18 | CONTROL | 51.27852 | 1.1729283 | 500m DS | 420 | 34 | 99 | 17 |
|  | IMPACT | 51.14163 | 1.283182 | 10m DS | 9 | 2 | 16 | 3 |
| 19 | CONTROL | 53.75978 | -2.0181037 | 230m US | 227 | 32 | 67 | 9 |
|  | IMPACT | 53.61996 | -2.1423 | 1400m US | 244 | 23 | 49 | 8 |
| 20 | CONTROL | 52.96757 | -1.5462825 | 670m US | 267 | 26 | 67 | 12 |
|  | IMPACT | 52.95 | -1.82974 | 520m DS | 302 | 28 | 56 | 4 |
| 21 | CONTROL | 54.2371 | -2.7239995 | 365m DS | 215 | 17 | 31 | 5 |
|  | IMPACT | 54.35067 | -2.75843 | 1035m DS | 215 | 14 | 31 | 5 |
| 22 | CONTROL | 52.87719 | -1.7934283 | 485m US | 296 | 40 | 58 | 4 |
|  | IMPACT | 52.95101 | -1.84371 | 25m US | 237 | 35 | 69 | 14 |
