# Supplementary material for: The effects of run-of-river hydroelectric power schemes on invertebrate community composition in temperate streams and rivers
Source: PLoS One. 2017 Feb 3;12(2):e0171634. doi: 10.1371/journal.pone.0171634 (PMC5291416; doi:10.1371/journal.pone.0171634)
Supplement: S3 Table — (DOCX) [file pone.0171634.s003.docx]

| **Invertebrate community metric** | **Treatment** | **Period** | **LS Mean** | **Lower CL** | **Upper CL** |
| --- | --- | --- | --- | --- | --- |
| Family richness | Control | Before | 20.45 | 17.83 | 23.08 |
|  |  | After | 19.75 | 17.31 | 22.19 |
|  | Impact | Before | 21.68 | 19.04 | 24.33 |
|  |  | After | 21.05 | 18.59 | 23.51 |
| Shannon-Wiener evenness index | Control | Before | 0.66 | 0.62 | 0.70 |
|  |  | After | 0.70 | 0.68 | 0.73 |
|  | Impact | Before | 0.69 | 0.65 | 0.73 |
|  |  | After | 0.68 | 0.65 | 0.70 |
| LIFE | Control | Before | 7.20 | 6.91 | 7.49 |
|  |  | After | 7.14 | 6.86 | 7.42 |
|  | Impact | Before | 7.18 | 6.89 | 7.47 |
|  |  | After | 7.14 | 6.86 | 7.43 |
| E-PSI | Control | Before | 77 | 64 | 86 |
|  |  | After | 75 | 62 | 85 |
|  | Impact | Before | 76 | 62 | 85 |
|  |  | After | 75 | 62 | 85 |
| WHPT-ASPT | Control | Before | 5.33 | 4.86 | 5.81 |
|  |  | After | 5.34 | 4.88 | 5.80 |
|  | Impact | Before | 5.41 | 4.94 | 5.89 |
|  |  | After | 5.43 | 4.97 | 5.90 |

**S3 Table. Least squares (LS) mean and 95% confidence limit (CL) for each treatment (Control-Impact) and period (Before-After), for the five invertebrate metrics.**
